# Use and effectiveness of PCSK-9 inhibitors in heart diseases: A review

**DOI:** 10.1097/MD.0000000000039308

**Published:** 2024-09-06

**Authors:** Nasser M. Alorfi, Maan H. Harbi, Maguy Saffouh El Hajj, Samha Alayoubi, Nihal Abdalla Ibrahim, Fakhr Z. Alayoubi

**Affiliations:** a Pharmacology and Toxicology Department, College of Pharmacy, Umm Al-Qura University, Makkah, Saudi Arabia; b College of Pharmacy, QU Health, Qatar University, Doha, Qatar; c Department of Mathematics and Sciences, College of Humanities and Sciences, Prince Sultan University, Riyadh, Saudi Arabia; d College of Pharmacy, Ajman University, Ajman, United Arab Emirates; e Center of Medical and Bio-Allied Health Sciences Research, Ajman University, Ajman, United Arab Emirates; f Department of Cardiac Sciences KFCC, King Khalid University Hospital, King Saud University, Riyadh, Saudi Arabia.

**Keywords:** Alirocumab, Clinical trials, Evolocumab, Heart diseases, PCSK-9 inhibitors

## Abstract

Proprotein Convertase Subtilisin/Kexin type-9 (PCSK-9) inhibitors have recently used in the management of different cardiac complications. Several clinical trials demonstrated their effectiveness in patients with hypercholesterolemia. However, the effectiveness of these medications in patients with heart diseases is still controversial. To review and summarize the clinical trials pertaining to the use and effectiveness of PCSK-9 inhibitors in heart diseases and to discuss the pharmacotherapy of these agents. A review was conducted of all clinical trials with PCSK-9 inhibitors for heart diseases registered at ClinicalTrials.gov since inception up to and including January 19th, 2024. These trials were retrieved. Data from these trials were extracted manually, categorized and analyzed. The number of identified clinical trials was 25,371. After screening and excluding irrelevant studies, 12 studies met the search criteria. The majority of these studies were conducted in the US. The total number of patients in these studies was 27,700. Alirocumab and Evolocumab were the most frequently used PCSK-9 inhibitors. This review identified only a few clinical trials on PCSK-9 inhibitors in heart disease patients. Therefore, it is recommended to conduct more randomized controlled clinical trials on PCSK-9 inhibitors in this patient population.

## 1. Introduction

Heart disease is a major global health issue and a leading cause of death in numerous countries, including the Middle East.^[1,2]^ Approximately 17.9 million people worldwide die from cardiovascular diseases (CVDs) each year.^[3]^ Heart attacks and stroke account for more than 4 out of 5 CVD deaths, and approximately 30% of these deaths occur prematurely in individuals under 70 years old.^[4]^

Several crucial behavioral risk factors significantly contribute to the development of heart diseases. Poor dietary choices, for example an excessive consumption of saturated and trans saturated fats and high intake of salt, and sugar, can cause obesity, hypertension, and dyslipidemia, all of which are key drivers of cardiovascular issues.^[5,6]^ Sedentary lifestyles, characterized by limited physical activity and prolonged periods of sitting, can also weaken the heart and the circulatory system over time.^[7,8]^ Furthermore, smoking is another major behavioral risk factor, as it damages blood vessels, reduces oxygen supply, and increases the likelihood of blood clots.^[9,10]^ Additionally, chronic stress and inadequate stress management can trigger inflammation, raise blood pressure, and strain the heart.^[11]^ Addressing these risk factors through behavioral interventions such as healthy eating habits, regular exercise, smoking cessation, and stress reduction techniques are crucial in preventing and managing heart diseases.

Proprotein Convertase Subtilisin/Kexin type-9 (PCSK-9) has arisen as a crucial player in the realm of CVDs. PCSK-9 functions by regulating cholesterol levels within the body.^[12]^ PCSK-9 is an enzyme produced primarily in the liver^[13]^ and attaches to the low-density lipoprotein (LDL) receptors on the outer part of liver cells.^[14]^ These receptors play a vital role in removing LDL cholesterol, also known as “bad cholesterol,” from the bloodstream. However, when PCSK-9 binds to these receptors, it triggers a process that leads to their degradation and reduces their ability to clear LDL cholesterol from the blood.^[14]^ This leads to increased levels of LDL cholesterol in the bloodstream, significantly raising the risk of CVDs.^[15]^ Inhibiting PCSK-9’s activity has become a therapeutic strategy to lower LDL cholesterol levels.^[16]^ Monoclonal antibodies targeting PCSK-9, when introduced into the body, can neutralize its function, allowing LDL receptors to remain active and effectively clear LDL cholesterol from the bloodstream. This approach has shown promise in managing elevated cholesterol levels and reducing the risk of heart diseases.^[17,18]^

### Aim:

To review and summarize the clinical trials pertaining to the use and effectiveness of PCSK-9 inhibitors in heart diseases and to discuss the pharmacotherapy of these agents.

## 2. Methods

### Data sources and search strategy:

ClinicalTrials.gov database was searched for clinical trials on the use of PCSK-9 inhibitors in heart diseases since inception up to and including January 19th, 2024. The search was conducted using “heart diseases” as the main keyword for conditions or diseases, with “PCSK-9” entered under the “other terms” section. A summary of the characteristics of the trials is obtained from the database, including the study title, conditions, interventions, phase, and number of patients enrolled.

### Data extraction:

From the results reported in the ClinicalTrials.gov registry, data were manually retrieved, and analyzed, according to the following elements:

- Interventions: Details of interventional (clinical trial), observational, and expanded access.
- Conditions: The keyword of condition/disease for searching was “heart diseases,” and PCSK-9.
- Trial design: All phases.
- Location: Open to any location.
- Study status: Completed and no longer looking for participants.

### Terms and synonyms searched:

The keywords or search terms used to find relevant clinical trials within the database were tabulated in Table 1.

**Table 1:** Terms and synonyms used in the search.

Terms	Search results	Entire database
Synonyms		
PCSK-9	67 studies	395 studies
PC9	–	3 studies
Heart diseases	67 studies	25,201 studies
Cardiac disease	–	328 studies
Cardiac disorder	–	18 studies
Cardiopathies	–	28 studies
Disease hearts	–	1 study
Diseases of the heart	–	3 studies
Disorder heart	–	1 study
Heart disorder	–	3 studies

## 3. Inclusion criteria

Completed and any phase clinical trials that were registered in the Clinicaltrials.gov database using PCSK-9 inhibitors as major intervention and related to heart diseases until January 19th, 2024 were included in this review. Age and sex/gender were not considered for excluding any clinical trial. Clinical trials that did not involve PCSK-9 inhibitors as a major intervention were excluded along with trials that did not have results.

## 4. Results

### Analysis of the number of research registrations:

A total of 25,371 registrations of clinical trials related to heart diseases were found in the ClinicalTrials.gov database. Records that were screened and studies that were completed were included. (n = 12,791). Of them, only 12 used PCSK-9 drugs met the inclusion criteria.

### Clinical trials characteristics:

Clinical trials characteristics including study title, conditions, interventions, phase, and number of enrolled patients are summarized in Table 2.

**Table 2:** Study title, conditions, interventions, phase and number enrolled (updated form ClinicalTrials.gov on October 19, 2024).

	Study title	Conditions	Interventions	Phase	Number of patients/participants enrolled	Location
1	Evolocumab in stable heart failure with reduced ejection fraction of ischemic etiology	Heart failure with reduced ejection fraction	Evolocumab	2	46	Spain
2	In-hospital initiation of PCSK-9 inhibitor in patients with acute myocardial infarction	Coronary disease	Evolocumab 140 mg/mL	NA	7556	NA
3	Effect of evolocumab on coronary endothelial function	HIV CAD	Evolocumab	2	19	United States
4	Evolocumab for early reduction of LDL-cholesterol levels in patients with acute coronary syndromes (EVOPACS)	Acute coronary syndrome	Evolocumab 140 mg/mL	3	308	Switzerland
5	Alirocumab and reverse cholesterol transport	Atherosclerosis CAD	Alirocumab	1	28	United States
6	Effects of acute, rapid lowering of LDL cholesterol with Alirocumab in patients With STEMI undergoing primary PCI	Acute coronary syndrome Hypercholesterolemia	Alirocumab	2	97	Canada
7	Alirocumab in patients with acute myocardial infarction	Myocardial infarction Hypercholesterolemia	Alirocumab	4	20	United States
8	Rosuvastatin and evolocumab for CAD	CAD	Statin Evolocumab	NA	195	United States
9	Effect of evolocumab on coronary plaque characteristic	CAD	Evolocumab Injections	4	137	United States
10	Imaging of coronary plaques in participants treated With evolocumab	CAD	Evolocumab Statin therapy	3	164	United States
11	ODYSSEY Outcomes: Evaluation of Cardiovascular Outcomes After an Acute Coronary Syndrome During Treatment With Alirocumab	Atherosclerotic cardiovascular disease	Alirocumab LMT	3	18924	United States
12	Evaluation of effect of Alirocumab on Coronary Atheroma Volume in Japanese Patients Hospitalized for Acute Coronary Syndrome With Hypercholesterolemia	Hypercholesterolemia Acute coronary syndrome	Alirocumab Atorvastatin Rosuvastatin	4	206	Japan

HIV = human immunodeficiency virus; LMT = lipid modifying therapy; CAD = coronary artery diseases; NA = not applicable.

### PCSK-9 drugs usage:

Two drugs have been used, Alirocumab and Evolocumab, as shown in Table 2.

### Prisma chart:

**Figure 1.**
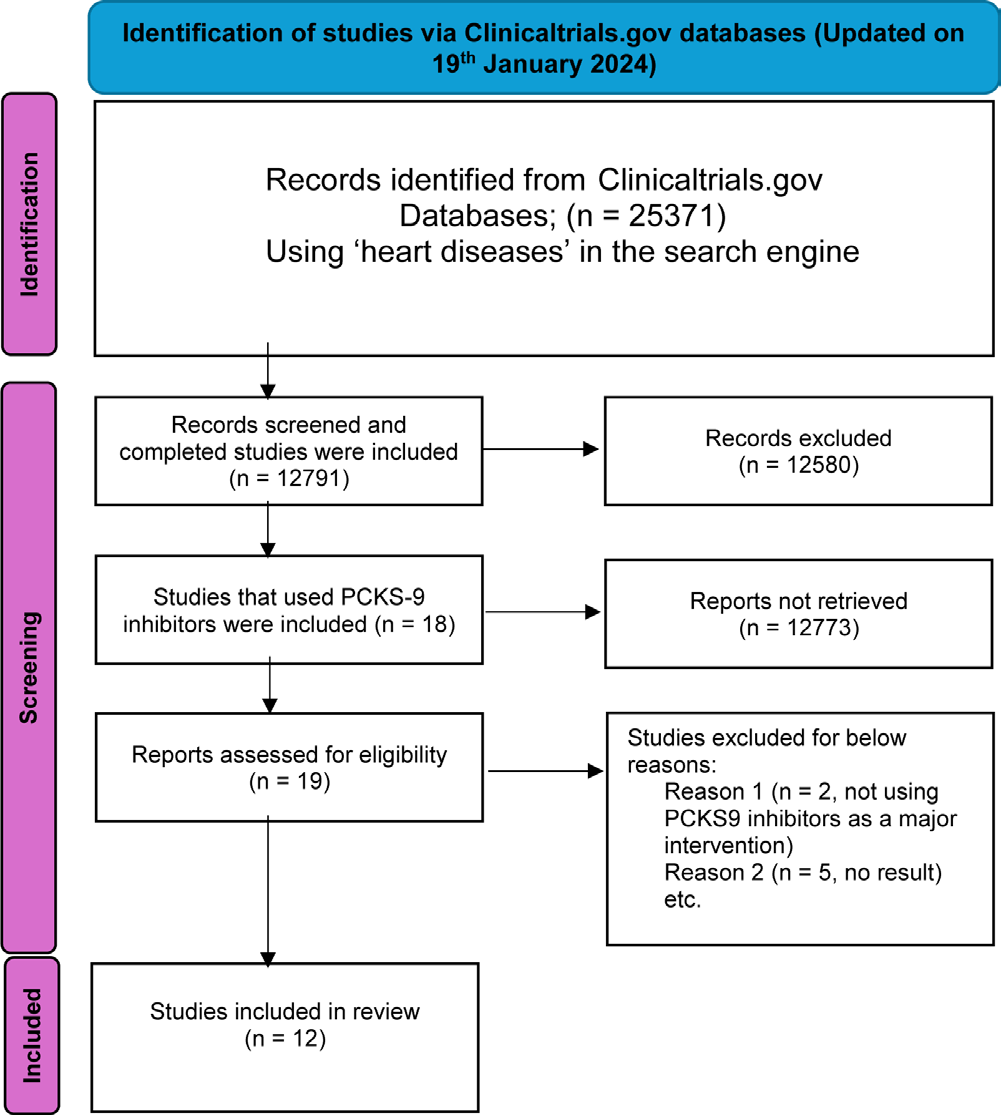
PRISMA flow diagram of the search identified from ClinicalTrials.gov database.

Figure 1 displays the identification of studies using the Clinicaltrials.gov database, updated on January 19, 2024.

## 5. Discussion

PCSK-9 inhibitors represent a promising class of lipid-lowering medications that have demonstrated a significant reduction in LDL-C levels and a decreased risk of major cardiovascular events in patients with heart disease. Utilization of PCSK-9 inhibitors in clinical practice is currently limited by their high cost and the lack of long-term safety data. It has been shown that Alirocumab is more effective than Evolocumab in high CV-risk patients whose LDL-C target goals were not met, while Evolocumab has stronger evidence in patients with heterogeneous familial hypercholesterolemia and patients with varied CV risks whose LDL-C target goals were not achieved.^[19]^

A monoclonal antibody, Alirocumab, binds to PCSK-9, preventing it from binding to LDL receptors on the surface of liver cells. This rises the number of LDL receptors on the surface of the liver cells, allowing more LDL cholesterol to be removed from the blood, thus lowering LDL cholesterol levels.^[20]^ A systematic review and meta-analysis of randomized clinical trials has demonstrated that Alirocumab significantly lowers LDL cholesterol levels by 45% to 62% compared to placebo, establishing it as an effective treatment for hypercholesterolemia.^[21]^ The Food and Drug Administration has approved Alirocumab for adults who have established cardiovascular disease, are at high risk for cardiovascular disease, or have familial hypercholesterolemia. Alirocumab has been proven to decrease the risk of major cardiovascular events such as heart attacks and strokes in patients with existing cardiovascular disease, hence its labeled indication of secondary prevention of cardiovascular events.^[17]^

Among the medications that have been developed, Evolocumab is a human monoclonal immunoglobulin G2 that binds specifically to human PCSK-9.^[22]^ It is used to lower cholesterol levels in patients with CVDs such as coronary artery disease, stroke, and peripheral artery disease.^[23]^ A systematic review and meta-analysis of randomized clinical trials has demonstrated that Evolocumab significantly decreases LDL cholesterol levels by 55% to 66% as compared to placebo, making it an effective treatment for hypercholesterolemia.^[24]^ Evolocumab received the Food and Drug Administration approval for its use in adults with established or at high risk of CVDs as well as in adult and pediatric patients older than 10 years of age with familial hypercholesterolemia. This is due to clinical evidence showing that Evolocumab effectively lowers LDL cholesterol, which is a key risk factor for CVDs.^[25]^

## 6. Long-term effects of using PCSK-9 inhibitors

PCSK-9 inhibitors are a relatively new class of drugs, and their long-term effects are not yet fully known. Yet, the existing evidence indicated that they are generally safe and well-tolerated in the short to medium-term.^[26]^

In clinical trials, PCSK-9 inhibitors have been shown to significantly reduce LDL cholesterol levels and to decrease the risk of cardiovascular events, such as heart attacks and strokes.^[17,18]^ However, the long-term effects on cardiovascular outcomes and mortality rates are still being studied. One concern with the long-term use of PCSK-9 inhibitors is the potential for adverse effects on the liver and kidneys. Some studies have suggested that these drugs may increase liver enzymes, which could indicate liver damage. However, the clinical importance of this result has not yet been established.^[17]^ Another potential concern is the development of anti-drug antibodies, which could diminish the effectiveness of these drugs over time. Nevertheless, this appears to be a relatively rare occurrence with PCSK-9 inhibitors.^[27]^ As with all medications, patients receiving PCSK-9 inhibitors should be monitored for potential side effects, and a careful evaluation of the benefits versus risks of the treatment should be conducted.

## 7. Conclusion

In conclusion, PCSK-9 inhibitors present a promising therapeutic avenue for heart patients, particularly those with elevated cholesterol levels and heightened cardiovascular risk. These inhibitors demonstrate notable effectiveness in significantly reducing LDL cholesterol levels, addressing a crucial risk factor for CVDs. By offering a potent adjunct to standard therapies, such as lifestyle modifications and statins, PCSK-9 inhibitors have demonstrated a potential in lowering the occurrence of major adverse cardiovascular events. However, personalized evaluation of patient profiles, considerations of cost and accessibility, and collaboration with healthcare providers remain essential in optimizing the use of these medications to enhance heart health outcomes.

## Acknowledgments

The author (SA) would like to thank Prince Sultan University, Riyadh for their support. The author (FA) would like to thank Saudi cardio clinical pharmacy group at Saudi Heart Association for their valuable support at this work.

## Author contributions

**Project administration:** Fakhr Z. Alayoubi.

**Resources:** Nasser M. Alorfi, Samha Alayoubi.

**Supervision:** Nasser M. Alorfi.

**Validation:** Nasser M. Alorfi, Maguy Saffouh El Hajj, Nihal Abdalla Ibrahim.

**Visualization:** Nasser M. Alorfi.

**Writing – original draft:** Nasser M. Alorfi, Fakhr Z. Alayoubi.

**Writing – review & editing:** Nasser M. Alorfi, Fakhr Z. Alayoubi, Maan H. Harbi, Maguy Saffouh El Hajj.
